# Clinical and prognostic usefulness of soluble urokinase plasminogen activator receptor in hemodialysis patients

**DOI:** 10.1007/s11255-017-1778-5

**Published:** 2018-01-08

**Authors:** Rafał Nikodem Wlazeł, Iwona Szadkowska, Piotr Bartnicki, Kinga Rośniak-Bąk, Jacek Rysz

**Affiliations:** 1 Department of Laboratory Diagnostics and Clinical Biochemistry, Medical University of Lodz, Lodz, Poland; 2 Department of Internal Diseases and Cardiological Rehabilitation, Medical University of Lodz, Lodz, Poland; 3 Department of Nephrology, Hypertension and Family Medicine, Medical University of Lodz, Lodz, Poland

**Keywords:** suPAR, Hemodialysis, Cardiovascular risk, Mortality risk

## Abstract

**Purpose:**

Considering its prognostic usefulness and the relationship with chronic kidney disease, we analyzed the clinical utility of soluble urokinase plasminogen activator receptor (suPAR) in end-stage renal disease patients undergoing hemodialysis treatment. We focused on the association between suPAR levels and clinical outcomes, especially those related to cardiovascular events and mortality as well as the effect of hemodialysis on the protein levels.

**Methods:**

We enrolled 64 patients. Blood samples for laboratory tests were collected before and after the midweek hemodialysis. The concentration of suPAR was assessed using suPARNostic ELISA, ViroGates.

**Results:**

Spearman rank analyses showed a positive association between suPAR and creatinine, cystatin C, galectin 3, N-terminal prohormone of brain natriuretic peptide and troponin T (*p* < 0.05). In ROC analysis, the suPAR concentration equal to 11.5 ng/mL was established to be the cutoff value for the prediction of mortality in the analyzed patients. Simultaneous analysis of creatinine and suPAR increased the predictive value of the latter—the area under curve increased to 0.84 (95% CI 0.70–0.94, *p* < 0.0001). Logistic regression analysis revealed that increase in the suPAR level was associated with the increase in odds ratio for death by 1.3 (95% CI 1.1–1.6, *χ*^2^ = 8.2, *p* = 0.004). In multivariable analysis, the prediction power of suPAR appeared to be stronger after including creatinine (*p* = 0.0005).

**Conclusions:**

Elevated suPAR levels provide independent information on mortality risk in patients undergoing hemodialysis. The protein appears not to cross the dialysis membrane; thus, blood collection before the second hemodialysis session seems to give reliable information on the suPAR level for clinical interpretation.

## Introduction

Soluble urokinase plasminogen activator receptor (suPAR) circulates in the human bloodstream and bodily fluids as a consequence of proteolytic cleavage of the glycosyl-phosphatidylinositol anchor of the urokinase plasminogen activator receptor that is expressed on membranes of various cells, including immunologically active cells and endothelial cells [1]. The protein is present in healthy individuals in a low concentration, while higher levels have been observed in persons with infectious diseases and other inflammatory disorders. Therefore, suPAR was considered to be a marker of immune system activation [1, 2]. The protein is involved in numerous signaling pathways, including those involved in cellular proliferation, migration, adhesion, and differentiation.

The level of soluble urokinase plasminogen activator receptor has been demonstrated to provide prognostic data concerning the risk of cardiovascular events and all-cause mortality in general population and critically ill patients [3, 4]. In the kidneys, the presence of the protein leads to proteinuria following podocyte migration and injury and is known as an important factor in the development of focal segmental glomerulosclerosis (FSGS) [1, 5]. Nevertheless, it was reported that in a population of patients with all-cause chronic kidney disease (CKD), suPAR correlated with reduced glomerular filtration rate (GFR) [5–7]. Considering its prognostic usefulness and the relationship with CKD, we decided to analyze the clinical utility of suPAR in end-stage renal disease patients. Due to the fact that heart failure (HF) is a leading cause of mortality in CKD [8, 9], we focused on the association between suPAR levels and clinical outcomes, especially those related to cardiovascular events and all-cause mortality. Additionally, we compared suPAR with other biomarkers with predictive value, such as N-terminal prohormone of brain natriuretic peptide (NT-proBNP), galectin-3 (Gal-3), high-sensitive troponin T (hsTnT), and high-sensitive C-reactive protein (hsCRP). The aim of this study was to analyze the prognostic potential of suPAR for predicting morbidity and mortality of HD patients as well as the effect of dialysis on the protein levels.

## Materials and methods

We enrolled 64 out of the 74 patients of the Department of Nephrology, Hypertension, and Family Medicine—Central Dialysis Unit of the Medical University of Lodz, Poland, undergoing hemodialysis treatment 3 times per week, excluding those with biopsy-proven FSGS. The additional excluding criteria were: malignancy, hepatic diseases, rheumatic and autoimmune diseases, and prior transplants. We enrolled stable HD patients only, already on dialysis for 4–73 months, median: 20 (6–56) months. All the enrolled patients had no symptoms of infection nor any other acute disease. The causes of CKD which progressed to end-stage renal disease in the group of the studied patients included: diabetic nephropathy (*n* = 27, 42%); hypertensive nephropathy (*n* = 13, 20%); interstitial nephritis (*n* = 9, 14%); obstructive nephropathy (*n* = 8, 12%); polycystic kidney disease (*n* = 5, 9%); and ischemic nephropathy (*n* = 2, 3%). Twenty (31%) of the patients had a history of coronary artery disease (CAD), while HF was diagnosed in 41 (64%) patients.

The patients were treated with HD in four-hour session using dialyzers based on polynephron membrane (Elisio-190M, Nipro, Osaka, Japan). All the patients had an arteriovenous fistula as vascular access. A single pool Kt/V was calculated according to the Daugirdas formula based on serum urea concentration before and after the midweek dialysis and before the next dialysis of the week to assess a dialysis adequacy. Blood flow and ultrafiltration rates were adjusted to the individual needs of the patients and kept constant.

Blood samples for laboratory tests were collected before and after the midweek session to determine the levels of laboratory markers, including NT-proBNP, Gal-3, hsTnT, hsCRP, cystatin C, urea, creatinine, albumin, total cholesterol, LDL, calcium, phosphate, parathormone (PTH), hemoglobin, ferritin, and total iron binding capacity (TIBC). Blood analysis before dialysis was performed to describe patients’ baseline characteristics, while the measurement of urea, suPAR, and albumin, also after dialysis, gave insight into how the dialysis session influenced the levels of those parameters. The concentration of suPAR was analyzed using suPARNostic ELISA, ViroGates, Denmark; Gal3 using enzyme-linked fluorescence assay (ELFA), bioMerieux, France; NT-proBNP and hsTnT using electro-chemiluminescence immunoassay (ECLIA), Roche, Switzerland; and cystatin C, hsCRP, immunoturbidimetric assay (ITA), Beckman Coulter, USA.

Heart failure (HF) was diagnosed according to HF criteria [10]. Transthoracic echocardiography was performed on inclusion to the study. Left ventricular ejection fraction (EF) was assessed by the modified Simpson’s formula. During a 3-year follow-up (2014-2016), we focused on clinical outcomes, including all-cause mortality, non-fatal cardiovascular events (myocardial infarction, stroke, hospitalization for heart failure), infections, and hospitalization for other reasons.

### Statistical analysis

All results, including baseline characteristics, are presented as mean ± standard deviation (SD) or median values with 5–95th percentiles, if necessary, for continuous variables and as percentages for categorical variables. Differences in analyzed parameters between studied groups of patients were analyzed using the Mann–Whitney test for independent samples and the Wilcoxon test for paired samples. The relation between the suPAR level and baseline characteristics was assessed using the Spearman or Pearson correlation analysis. A comparison of appropriate parameter levels between groups, with and without the endpoints, was performed with the Kruskal–Wallis test. Logistic regression models were used for the analysis of the relations between determined laboratory biomarkers and patients’ outcomes during the follow-up period. Forward selection was used. Additionally, a receiver operating characteristic (ROC) curve analysis was performed to determine the predictive value of biomarkers as well as to define their optimal cutoff values. The statistical analysis was performed using MedCalc Statistical Software version 17.5.5 (MedCalc Software bvba, Ostend, Belgium; http://www.medcalc.org; 2017). The significance level of *p* < 0.05 was used in all tests.

## Results

The study group consisted of 64 persons (42 men and 22 women) with a mean age of 66 ± 13 years. The mean serum concentration of suPAR measured before hemodialysis was 14.6 ± 6.0 ng/mL. When performing the Spearman rank and Pearson analyses, we found a positive association between suPAR concentrations and creatinine, cystatin C, Gal-3, NT-proBNP, and hsTnT, as well as the dialysis vintage (*p* < 0.05). We also observed a negative association with serum albumin and cholesterol; however, these associations were rather weak (− 0.37; *p* = 0.022 and − 0.30; *p* = 0.058, respectively). There was no correlation between suPAR level and others among known associations (Table 1).

**Table 1:** Correlation table between suPAR serum concentration and baseline characteristics of the studied group

Variable	*r*	*p* value
Age	− 0.12	0.45
Gender (male vs. female)	− 0.16	0.32
Diabetes mellitus	− 0.004	0.98
Dialysis vintage	**0.399**	**0.0089**
Heart failure	**0.55**	**0.0002**
Coronary artery disease	**0.45**	**0.0026**
Atrial fibrillation	0.056	0.72
EF	− 0.3	0.05
NT-proBNP	**0.54**	**0.0003**
Galectin 3	**0.44**	**0.0038**
hsTnT	**0.52**	**0.0005**
hsCRP	0.23	0.15
Cystatin C	**0.36**	**0.02**
Creatinine	**0.33**	**0.038**
Albumin	− **0.37**	**0.022**
Cholesterol	− 0.30	0.058
LDL	− 0.21	0.20
Calcium	0.18	0.26
Phosphate	0.13	0.43
Parathormone	0.12	0.48
Hemoglobin	0.004	0.98
Ferritin	− 0.43	0.79
Total iron binding capacity	− 0.19	0.23

Pearson (parametric) or Spearman rank (nonparametric) analysis was performed, as appropriate

Statistically relevant correlations are shown in bold

Abbreviations used: *EF* (left ventricular) ejection fraction, *NT*-*proBNP* N-terminal prohormone of brain natriuretic peptide, *Gal*-*3* galectin-3, *hsTnT* high-sensitive troponin T, *hsCRP* high-sensitive C-reactive protein, *LDL* low-density lipoprotein (cholesterol)

Significantly higher suPAR concentrations were observed in all patients with a history of cardiovascular disease (17.5 vs. 12.6 ng/L; *p* = 0.004). Additionally, patients with a history of CAD had considerably higher suPAR levels (14.9 vs. 9.6 ng/L; *p* = 0.012). Despite the observed differences between baseline suPAR level in patients with and without diagnosed HF (15.1 vs. 9.1 ng/mL; *p* = 0.0004), there were no differences in the suPAR level between HF patients with preserved and reduced EF. There were no differences between suPAR levels in patients with and without atrial fibrillation (14.6 vs. 13.2 ng/L; *p* = 0.7).

During the follow-up period, we observed 15 non-fatal cerebrocardiovascular events (2 myocardial infarctions, 4 strokes, 4 hospitalizations for decompensation of heart failure, 5 peripheral arteries angioplasty procedures) and 37 hospitalizations for other reasons. We observed statistically relevant differences in baseline suPAR level in patients who finally reached endpoint (14.7 vs. 11.2 ng/mL, *p* = 0.043), and the ROC analysis showed the predictive power of the protein in the prediction of cerebrocardiovascular endpoint (AUC = 0.7, *p* = 0.029). Nevertheless, these data were not statistically relevant in uni- or multivariate regression analysis (*p* > 0.05).

During the follow-up period, 33 (51%) patients died. Due to the low number of deaths of cardiovascular cause (*n* = 12), we analyzed the relationship between suPAR and all-cause mortality. The logistic regression analysis performed in a forward stepwise manner revealed that a 1 ng/mL increase in suPAR level was associated with an increase in OR for death by 1.3 (95% CI 1.1–1.6, *χ*^2^ = 8.2, *p* = 0.004). A statistically relevant relationship was also found in the case of albumin concentration. An increase in albumin concentration by 1 g/L correlated with a decrease in OR for death by 0.6 (95% CI 0.5–0.9, *χ*^2^ = 13.5, *p* = 0.0002). In multivariable analysis, the prediction power of suPAR appeared to be stronger after including creatinine concentrations (*χ*^2^ = 15.3, *p* = 0.0005). Known risk factors, like the patients’ age, dialysis vintage, and diabetes, as well as other biomarkers in question: NT-proBNP, Gal-3, hsCRP, and hsTnT, were also included in the analysis, but they did not retain in the regression model (*p* > 0.1).

ROC analysis enabled the assessment of suPAR usefulness as the predictor of mortality in the analyzed patients. A suPAR concentration equal to 11.5 ng/mL was established to be the best-fit cutoff value with 90% sensitivity but only 53% specificity (*p* = 0.006). Nevertheless, the positive (PPV) and negative (NPV) predictive values, assuming that the ratio of cases in the positive and negative groups reflects the prevalence of outcomes, were 72 and 82%, respectively. Therefore, we used this concentration level as an optimal criterion to determine a division into patient groups for the further analysis. The clinical and laboratory characteristics of patients with or without elevated suPAR levels are presented in Table 2.

**Table 2:** Baseline characteristics of the studied group

Variable	Overall (*n* = 64)	suPAR ≤ 11.5 ng/L (*n* = 21)	suPAR > 11.5 ng/L (*n* = 43)	*p* value
Age (year)	66.7 ± 13	65.9 ± 13.2	67.3 ± 15.7	0.42
Gender: male versus female [*n* (%)]	42 versus 22 (66 vs. 34)	16 versus 4 (85 vs. 15)	24 versus 20 (55 vs. 45)	0.06
Coronary artery disease [*n* (%)]	20 (31)	2 (10)	18 (90)	0.03
Atrial fibrillation [*n* (%)]	17 (26)	2 (12)	15 (88)	0.07
Heart failure [*n* (%)]	41 (66)	5 (12)	36 (88)	0.0001
Diabetes [*n* (%)]	30 (47)	12 (40)	18 (60)	0.43
EF (%)	46 ± 10	44 ± 9	52 ± 9	0.012
NT-proBNP (pg/mL)	6881 (91–35,000)	1233	11,161	0.007
Galectin 3 (ng/mL)	55.3 ± 25.8	58.5	48.3	0.24
hsTnT (ng/L)	60.8 (16.2–199.5)	36.2	81.9	0.012
hsCRP (mg/L)	4.8 (4.8-45.5)	4.8	5.1	0.69
Cystatin C (mg/L)	4.9 ± 0.7	4.4	5.1	0.03
Creatinine (μmol/L)	644 ± 238	570	678	0.18
Albumin (g/L)	38.6 (14.7-45.0)	42.2	38.1	0.017
Total cholesterol (mmol/L)	4.16 ± 1.00	4.67	3.96	0.002
LDL (mmo/L)	2.62 ± 0.84	2.97	2.25	0.09
Calcium (mmol/L)	2.21 ± 0.18	2.16	2.23	0.20
Phosphate (mmol/L)	1.79 ± 0.58	1.64	1.88	0.21
Parathormone (pmol/L)	33.8 ± 23.8	30.6	35.6	0.56
Hemoglobin (g/dL)	10.7 ± 1.5	10.7	10.8	0.91
Ferritin (ng/mL)	798 ± 574	836	756	0.68
Total iron binding capacity (μmol/L)	42 ± 8	43	41	0.42
suPAR (ng/mL)	14.6 ± 6.0	8.9	17.3	<0.0001

Data are expressed as mean or median as appropriate. Groups are categorized according to the best-fit suPAR cutoff value in prediction of mortality. Differences between groups are tested using parametric *T* test, Mann–Whitney or Chi-square test, as appropriate. Explanations of the abbreviations are provided with Table 1

**Fig. 1:**
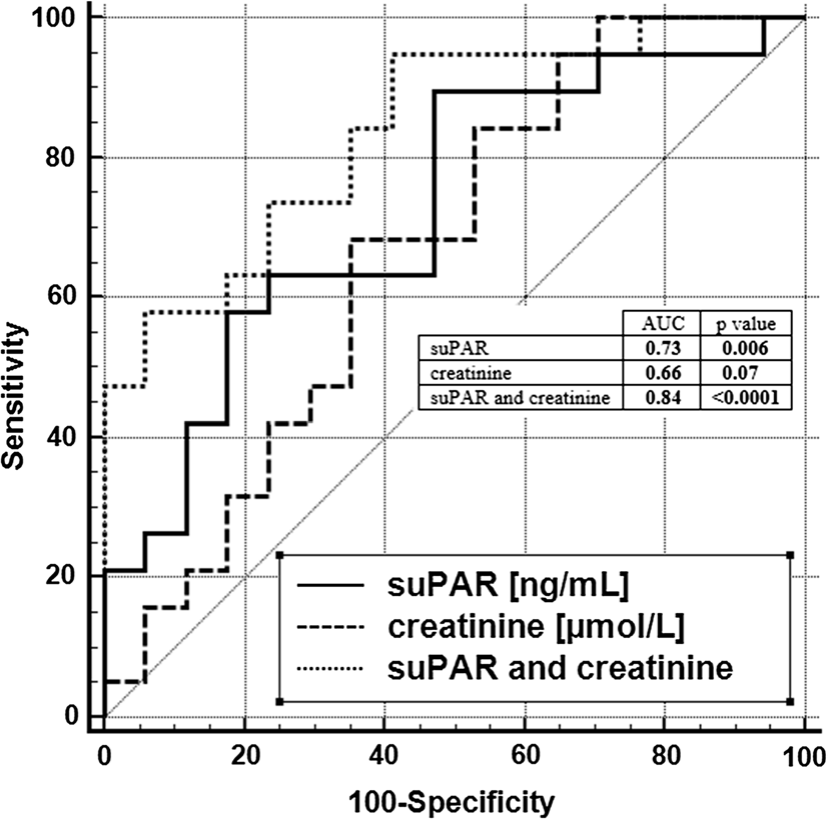
The results of the comparative ROC analysis in prediction of mortality in studied patients

**Fig. 2:**
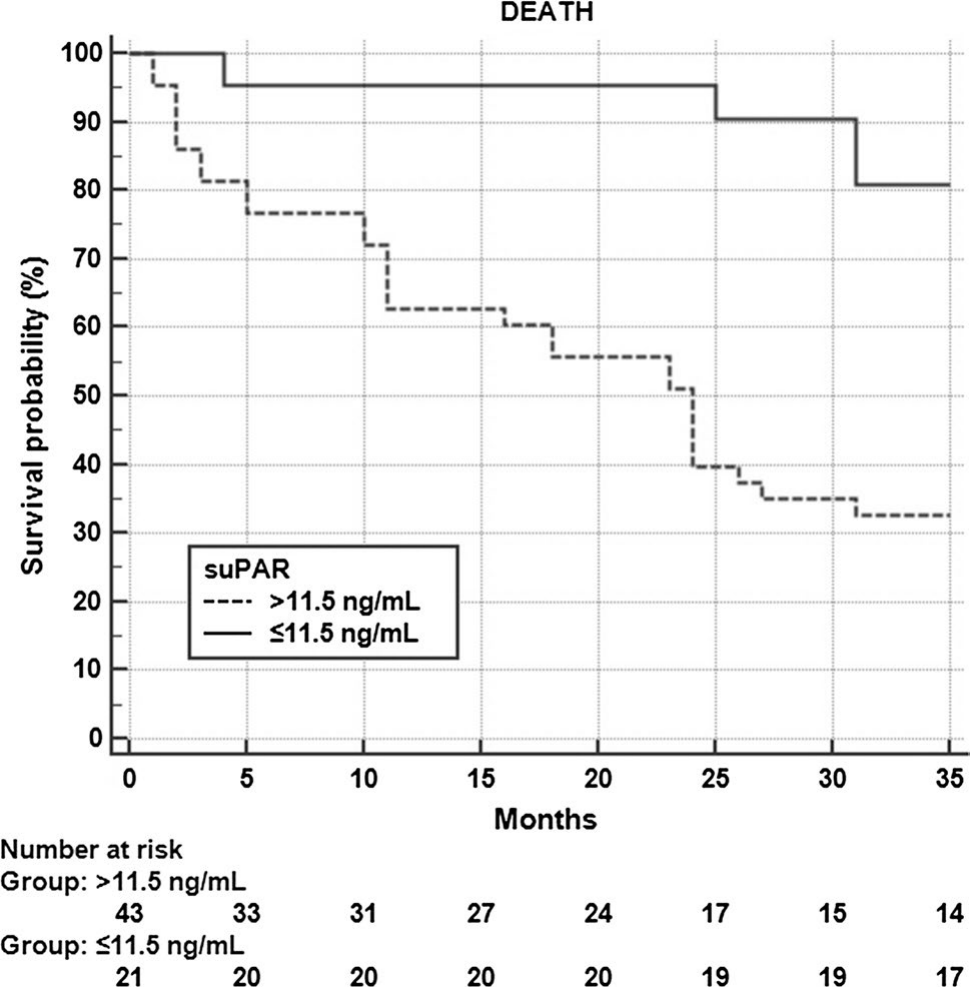
Kaplan-Meier survival analysis—comparison between groups with suPAR lever higher and lower than the best-fit cutoff value in prediction of mortality

Although the predictive value of creatinine levels was shown to be of borderline relevance in the ROC analysis (AUC = 0.66, *p* = 0.079), simultaneous analysis of creatinine and suPAR increased the predictive value of the latter—the AUC of suPAR increased to 0.84 (95% CI 0.70–0.94, *p* < 0.0001). The comparative ROC analysis is presented in Fig. 1. These results are in accordance with the correlation analysis and the logistic regression analysis presented above. Additionally, Kaplan–Meier survival analysis showed a 5.6 (95% CI 2.3–13.8) times higher hazard ratio of mortality in the group of patients with suPAR level higher than 11.5 ng/mL; *p* = 0.007 (Fig. 2).

We observed differences in the numerical values of suPAR serum concentrations before and after hemodialysis session in individual patients (means 14.6 vs. 14.8 ng/mL, respectively) and the differences in the protein level ranged between 1.7 and 26.8%. Nevertheless, the paired-samples *t* test showed no statistical relevance of the differences (*p* = 0.1), while there was a high correlation between the concentrations (*r* = 0.92, *p* < 0.0001). Due to the fact that there are no data concerning suPAR concentration following dialysis, we tried to assess whether its concentration changes as a result of going through dialysis membranes. We compared the suPAR (60 kDa) level changes with albumin (66 kDa) level changes (the easily detectable protein that does not cross the barrier, with a similar molecular mass) and the urea reduction rate (URR). The median concentrations of albumin before and after hemodialysis session were 38.6 and 43.5 g/L, respectively, and the differences in the protein levels ranged between 1.7 and 22.5%. The URR ranged from 0.52 to 0.88 with the mean 0.65 ± 0.06. We found a correlation between changes in suPAR levels and changes in albumin levels (*p* = 0.001) but no correlation with URR (*p* = 0.4).

## Discussion

The mortality risk of patients undergoing hemodialysis is undisputedly high and frequently remains in association with cardiovascular complications [11]. In this study, we observed a significantly higher baseline suPAR level in patients with diagnosed HF and in patients with a history of cardiovascular disease. ROC analysis showed the predictive power of suPAR protein in the prediction of cerebrocardiovascular endpoint (AUC = 0.7, *p* = 0.03). Meijers et al. [12] in their study also showed that suPAR level was directly and significantly associated with cardiovascular events in a group of 476 patients with mild-to-moderate kidney disease. However, in contrast to this study, we failed to find statistically relevant association with CV events in our patients, while, what is worth to point, the mean suPAR concentration in this group of the patients was already significantly higher. Additionally, in the population without CKD, elevated plasma suPAR levels were associated with the presence and severity of angiographic CAD. In Eapen et al.’s [13] study, suPAR levels were higher in patients with significant CAD compared to those with normal coronary arteries or insignificant CAD, and a greater severity of CAD was associated with higher suPAR levels.

In our multivariate analysis, only suPAR, creatinine, and hypoalbuminemia were associated with mortality. We found that a 1 ng/mL increase in suPAR level was associated with an increase in OR for death by 1.3. Moreover, only suPAR appeared to give independent information for risk stratification in the studied patients against other analyzed parameters: NT-proBNP, Gal-3, hsCRP, and hsTnT. Eapen et al. [13] confirmed that suPAR was a significant predictor of incident mortality and morbidity in patients with suspected or established CAD. Moreover, in their study, suPAR significantly improved discrimination of future death and MI risk over a standard clinical model.

Our study confirmed the known relationship between albumin concentration and mortality. The increase in albumin concentration by 1 g/L correlated with a decrease in OR for death by 0.6 (95% CI 0.5–0.9, *χ*^2^ = 13.5, *p* = 0.0002). In other studies, similar associations were observed [14–17].

It was previously proved that suPAR levels are elevated in association with cardiovascular disease and they hold potential information concerning short- and long-term cardiovascular prediction [2, 3, 18]. Many other markers have also recently been studied in the context of cardiovascular prediction, including galectin-3, NT-proBNP, and troponin T, among others [19–21]. Additionally, in the context of cardiovascular risk in dialysis patients, markers of cardiac dysfunction were recently studied [22, 23]. The results of our studies show a correlation between suPAR and the markers of heart failure and heart injury. A suPAR concentration equal to 11.5 ng/mL was established to be the cutoff value with 90% sensitivity, but only 53% specificity (*p* = 0.006). This relatively low specificity may be due to the fact that the calculated optimal level is much above the concentration recognized as associated with a moderate risk (6 ng/mL) and a high risk (9 ng/mL) in a particular patient and associated with critical illness anyway [3, 4]. Though high concentration of creatinine in the studied group of patients is a complex consequence of nutrition, HD adequacy and residual kidney function, simultaneous analysis of creatinine and suPAR increased the predictive value of the latter—the AUC of suPAR increased to 0.84 (95% CI 0.70–0.94, *p* < 0.0001). Additionally, Kaplan–Meier survival analysis showed a 5.6 times higher hazard ratio of mortality in the group of patients with suPAR level higher than 11.5 ng/mL (*p* = 0.007). Only suPAR fit the final model of the risk estimation, which made the biomarker an independent predictor of overall mortality in the studied group. Although a past history of cardiovascular disease was associated with mortality in our patients, we could not study associations between the markers in question and mortality due to cardiovascular events only because of the relatively small number of events.

Due to the fact that there are no data concerning suPAR concentration following dialysis, we also tried to assess whether its concentration changes as a result of going through dialysis membranes. A strong correlation between suPAR level changes and albumin level changes measured before and after the hemodialysis session and no association with URR proves that the molecular form of the protein in question appears not to cross the dialysis membrane: thus, blood collection before the second hemodialysis session seems to give reliable information on the suPAR level for clinical interpretation. We concluded that the observed serum suPAR level changes during hemodialysis session are not due to crossing the hemodialysis membrane, but are the results of changes in the volume of bodily fluids (dehydration after the session) and/or other influences of the preanalytical phase.

The limitations of this study include the relatively small study group and the fact that it enrolled HD patient only from one dialysis unit. Nevertheless, the mean concentration of suPAR in our studied group (14.6 ng/mL) was in accordance with the data obtained by Griveas et al. [24], who described the same mean concentration of the marker in question (14.7 ng/mL) in a similar group, but which was twice as big (*n* = 127) of HD patients.

## Conclusions

Elevated suPAR levels provide independent information on all-cause mortality risk in patients undergoing hemodialysis. The protein appears not to cross the dialysis membrane; thus, blood collection before the midweek hemodialysis session seems to give reliable information on the suPAR level for clinical interpretation.
